# Quantitative Analysis of Interaction Between CADM1 and Its Binding Cell-Surface Proteins Using Surface Plasmon Resonance Imaging

**DOI:** 10.3389/fcell.2018.00086

**Published:** 2018-08-07

**Authors:** Takeshi Ito, Yutaka Kasai, Yuki Kumagai, Daisuke Suzuki, Misaki Ochiai-Noguchi, Daisuke Irikura, Shiro Miyake, Yoshinori Murakami

**Affiliations:** ^1^Division of Molecular Pathology, The Institute of Medical Science The University of Tokyo, Tokyo, Japan; ^2^Bio/Life Science Team, Advanced R&D Center HORIBA Ltd., Kyoto, Japan

**Keywords:** surface plasmon resonance imaging, protein–protein interaction, cell adhesion molecule, immunoglobulin superfamily, adult T-cell leukemia

## Abstract

The cell adhesion molecule (CADM) family of the immunoglobulin superfamily (IgSF) comprises four members, CADM1–CADM4, and participates in the formation of epithelial and synaptic adhesion through cell–cell homophilic and heterophilic interactions. To identify the partners that interact with each member of the CADM family proteins, we set up a platform for multiple detection of the extracellular protein–protein interactions using surface plasmon resonance imaging (SPRi) and analyzed the interactions between the CADM family proteins and 10 IgSF of their structurally related cell adhesion molecules. SPRi analysis identified a new interaction between CADM1 and CADM4, where this heterophilic interaction was shown to be involved in morphological spreading of adult T-cell leukemia (ATL) cells expressing CADM1 when incubated on CADM4-coated glass. Moreover, class-I MHC-restricted T-cell-associated molecule (CRTAM) was identified to show the highest affinity to CADM1 among its binding partners by comparing the dissociation constants calculated from the SPR sensorgrams. These results suggest that the SPRi platform would provide a novel screening tool to characterize extracellular protein–protein interactions among cell-surface and secreted proteins, including IgSF molecules.

## Introduction

The immunoglobulin superfamily (IgSF) encompasses cell-surface and secreted proteins with immunoglobulin loops that are involved in a variety of physiological functions, including immune regulation, neural transmission, and epithelial tissue formation through interaction with IgSF molecules, integrins, and other proteins within the extracellular space (Aricescu and Jones, 2007). Most of IgSF molecules form both homophilic and heterophilic interactions to perform their functions; however, because of the limited approaches to identifying the partners in these interactions, their interaction network is not fully understood (Gonzalez, 2012). For example, immunoprecipitation coupled with mass spectrometry is generally not applicable for detecting extracellular interactions because of the low affinity of the membrane proteins. The yeast two-hybrid screening system is also unsuitable because extracellular proteins are often misfolded in the nucleus. Furthermore, comprehensive screening of molecular interaction using synthesized proteins from *Escherichia coli* or the wheat germ cell-free system is not appropriate for detecting interactions between extracellular proteins, like IgSF molecules (Wright et al., 2010) because post-translational modification such as glycosylation, which is critical to extracellular interactions, cannot be reconstituted.

A number of methods have been developed to screen the interactions between a pair of IgSF molecules. One such method is the avidity-based extracellular interaction screen (AVEXIS), a type of ELISA, used to detect the direct interactions between each bait–prey pair of recombinant protein fragments. AVEXIS was used to screen pairwise interactions between 249 of cell-surface and secreted IgSFs and leucine-rich repeat proteins from a protein library of a zebrafish (*Danio rerio*) and a number of molecular interactions were newly identified (Bushell et al., 2008; Martin et al., 2010). Similar findings were obtained for the interactions between IgSF, leucine-rich repeat, and fibronectin type III proteins in *Drosophila melanogaster* using the extracellular interactome assay, a modified AVEXIS method (Özkan et al., 2013). The protein microarray assay also succeeded in detecting several interaction pairs by screening a set of 89 human IgSF proteins against 686 highly diverse extracellular proteins (Ramani et al., 2012).

Surface plasmon resonance (SPR) is a label-free and real-time bio-sensing technology that is widely used for the validation of direct protein–protein interactions. Although SPR requires micrograms of purified proteins and so is not generally suitable for large-scale screening of protein–protein interactions, it was used to screen >2,000 proteins to identify the co-receptor for the B- and T-lymphocyte attenuator (BTLA), a kind of immune receptor of IgSF (Gonzalez et al., 2005). The interactions between 36 IgSF proteins and leucocyte surface proteins was examined using a 6 × 6 SPR interaction array (Jiang and Neil Barclay, 2010). Recently, SPR imaging (SPRi) instruments have been developed carrying a larger sensoring surface than conventional SPR to enable multiple detection of protein–protein interactions in combination with microarray spotting technology (Faye et al., 2009).

Here we developed a platform for screening the pairs of interacting IgSF molecules using a SPRi system. Our platform can detect up to 96 protein interactions in a single injection and the amount of protein solution required for spotting onto the chip surface is <10 nL, making it conducive to large-scale screening. Using the SPRi system, we examined the interactions among the IgSF molecules, including the cell adhesion molecule (CADM) and the Nectin families. The CADM and the Nectin families comprise four members each and share three Ig loops within the extracellular region and a short cytoplasmic domain. These molecules are involved in various types of cell–cell adhesion, such as that between epithelial cells or synaptic cells through homophilic and heterophilic interactions within the family molecules (Takai et al., 2008). Poliovirus receptor (PVR) is structurally related to the CADM and the Nectin families and forms heterophilic interactions with CADM1 and Nectin-3 (Ikeda et al., 2003; Wakayama et al., 2007). CADM1 also forms a heterophilic interaction with class-I MHC-restricted T-cell-associated molecule (CRTAM), another IgSF molecule (Arase et al., 2005; Boles et al., 2005; Galibert et al., 2005). By producing recombinant proteins of 10 IgSF molecules (CADM1–CADM4, Nectin-1–Nectin-4, PVR, and CRTAM), we examined their interactions with the CADM family proteins using SPRi.

## Materials and methods

### Cells

FreeStyle 293-F cells were purchased from Thermo Fisher Scientific (Waltham, MA, USA) and cultured in FreeStyle 293 Expression Medium (Thermo Fisher Scientific) supplemented with 100 units/mL penicillin and 100 μg/mL streptomycin (Thermo Fisher Scientific). 293FT cells were purchased from Thermo Fisher Scientific and cultured in Dulbecco's modified Eagle's medium with 4.5 g/L glucose (Nacalai Tesque, Kyoto, Japan) supplemented with 10% fetal bovine serum (FBS) (BioWest, Nuaille, France), 100 units/mL penicillin, and 100 μg/mL streptomycin. ATN1 cells were obtained from RIKEN BioResource Center (Tsukuba, Japan), cultured in RPMI1640 medium (Nacalai Tesque) supplemented with 10% FBS, 100 units/mL penicillin, and 100 μg/mL streptomycin, and the cells were maintained at 37°C in a humidified 5% CO_2_ incubator (Panasonic, Osaka, Japan).

### Protein production

The FreeStyle™ 293 Expression System (Thermo Fisher Scientific) was used to produce IgSF-Fc protein, and the expression vectors for protein production were prepared using Gateway™ Cloning Technology (Thermo Fisher Scientific), as described below.

#### Vectors

Extracellular domains of the 10 IgSF molecules, CADM1 (NP_055148; 1–374 a.a.), CADM2 (NP_001161146; 1–367 a.a.), CADM3 (NP_001120645; 1–330 a.a.), CADM4 (NP_660339; 1–324 a.a.), Nectin-1 (NP_002846; 1–355 a.a.), Nectin-2 (NP_002847; 1–360 a.a.), Nectin-3 (NP_056295; 1–404 a.a.), Nectin-4 (NP_112178; 1–349 a.a.), PVR (NP_006496; 1–343 a.a.), and CRTAM (NP_062550; 1–287 a.a.), were cloned into the pENTR/D-TOPO vector (Thermo Fisher Scientific). To obtain a Gateway-based destination vector, a Gateway cassette containing *attR* recombination sites flanking a *ccdB* gene and a chloramphenicol-resistance gene was cloned into the SmaI site of pHEK293 Ultra Expression Vector II (TaKaRa Bio Inc., Shiga, Japan), and a human IgG2-Fc fragment from the pFUSE-hIgG2-Fc1 vector (InvivoGen, San Diego, CA, USA) was subsequently inserted into the vector's XhoI-SphI site. This destination vector was named “pHEK-Fc.” The expression vectors of the extracellular domains of the IgSF molecules with a C-terminal Fc tag were obtained using a Gateway recombination with LR clonase (Thermo Fisher Scientific) between the pENTR/D-TOPO and pHEK-Fc vectors.

#### Transfection

For the production of IgSF-Fc proteins, 30 μg of each expression vector and 6 μg pHEK293 Enhancer Vector (TaKaRa Bio Inc.) were transiently transfected into 3 × 10^7^ FreeStyle 293-F cells with 36 μL TransIT-PRO Reagent (Mirus Bio, Madison, WI, USA) in 30 mL Freestyle 293 Expression Medium (Thermo Fisher Scientific). The cells were cultured in a disposable Erlenmeyer flask (Thermo Fisher Scientific) that was shaken at 120 rpm using a rotary shaker (RemoteShake; Wakenbtech, Kyoto, Japan) for 5 d at 37°C in a humidified 8% CO_2_ incubator.

#### Protein purification

The supernatants from 293-F cells transfected with IgSF-Fc expression vectors were filtered through a 0.45-μm membrane filter (Merck Millipore, Darmstadt, Germany). Prewashed 50 μL of Protein G Sepharose (GE Healthcare, Little Chalfont, UK) was added to each supernatant and rotated overnight at 4°C. Protein G Sepharose was collected after brief centrifugation and placed into MicroSpin Empty Columns (GE Healthcare). After five washes with 400 μL wash buffer (50 mM Tris-HCl, 150 mM NaCl, pH 7.5), the proteins were eluted by adding 200 μL elution buffer (0.1 M glycine, pH 3.0) and were subsequently neutralized with 30 μL 1 M Tris-HCl (pH 8.8). This elution step was performed twice. The obtained protein solution was dialyzed against phosphate buffered saline (PBS) using a Slide-A-Lyzer MINI Dialysis Device (Thermo Fisher Scientific). Each protein was stained with silver, and protein purity was analyzed using the Silver Stain MS Kit (Wako Pure Chemical Industries, Osaka, Japan). The Bradford method, the Bio-Rad Protein Assay Kit (Bio-Rad, Hercules, CA, USA), and a microplate reader (Model 680; Bio-Rad) were used to measure protein concentration. If needed, the protein solution was concentrated using Amicon Ultra Centrifugal Filter Units (Merck Millipore). We obtained ~10–700 μg purified protein from 30 mL of the supernatant from 293-F cells that were transiently transfected with the expression vectors.

### Surface plasmon resonance

Binding assays were performed using the SPRi system (OpenPlex; HORIBA France, Palaiseau, France) according to instructions in the previous report (Yamasaki et al., 2016) with some modifications.

#### Preparation of sensor chip

For the preparation of sensor chips, purified IgSF-Fc proteins and normal human IgG (Sigma-Aldrich, St. Louis, MO, USA) in PBS were spotted onto an SPRi-Biochip (HORIBA France) at 10 nL/spot using a dedicated spotter in a 96-spot format at HORIBA Ltd. (Kyoto, Japan). The spotted proteins were immobilized on the chip by amine coupling. The prepared sensor chip was inserted into the instrument according to the manufacturer's instructions.

#### Conditions for binding assays

All the reactions were performed at 25°C. Running buffer [PBS with 0.2% bovine serum albumin (BSA) and 0.02% Tween20] continuously flowed into the reaction chamber at 50 μL/min using the MINIPULS 3 peristaltic pump (Gilson, Inc., Middleton, WI, USA). The prepared protein samples were first preserved in a sample loop (volume = 200 μL) and then allowed to flow into the chamber by switching the valve to the injection mode. Intensity of the light reflected from an 810-nm LED with attenuation by SPR phenomena was recorded every 3 s using an 8-bit charge-coupled device (CCD) camera. The reflectivity of each spot was altered when the protein samples or cells were bound to the immobilized proteins. Percent change in reflectivity (%ΔR) was calculated on the basis of the CCD signal and normalized by subtracting the reflectivity for the same concentration of normal human IgG. Data were processed and analyzed using ScrubberGen (HORIBA France). A schematic diagram of the SPRi experiment is shown in Supplementary Figure 1.

#### Regeneration of the sensor chip

Most of the interactions among the purified proteins were dissociated spontaneously within 30 min by the continuous flow of running buffer without any regeneration reagents. If needed, 0.1 M acetic acid was injected into the chamber for 2 min at 25 μL/min and flushed out with running buffer at 1 mL/min. Removal efficiency was monitored by SPR signals and the sensor chip was reused for the next binding assay without any other treatments after the signals had sufficiently returned to baseline levels.

### Antibodies

Mouse monoclonal anti-FLAG M2 and anti-GFP antibodies were purchased from Sigma-Aldrich and Roche (Basel, Switzerland), respectively. Rabbit polyclonal anti-CADM1 (C-18) antibody was previously described (Ito et al., 2011). Chicken monoclonal anti-CADM1 antibody (9D2) was purchased from Medical and Biological Laboratories Co., Ltd (Nagoya, Japan). Goat polyclonal anti-GAPDH (V-18) was purchased from Santa Cruz Biotechnology (Santa Cruz, CA, USA). Goat polyclonal anti-human IgG (Fc specific) antibody was purchased from Sigma-Aldrich.

### Immunoprecipitation

293FT cells were transfected with CADM4-FLAG, CADM1-GFP, CADM3-GFP, and E-cadherin-GFP in four separate 6-cm dishes. After 24 h, the cells transfected with CADM4-FLAG were co-cultured with cells transfected with CADM1-GFP, CADM3-GFP, or E-cadherin-GFP in 10-cm dishes at a 1:1 ratio. After 2-day culture, the co-cultured cells were treated with 1 mM DTSSP/PBS for 30 min at room temperature, and any cross-linking was quenched with 20 mM Tris-HCl (pH 7.4). The cells were lysed with TNE buffer (10 mM Tris-HCl, 150 mM NaCl, 1 mM EDTA, 1% Triton-X100, pH 7.8) containing a protease inhibitor cocktail (200 μM AEBSF, 10 μM leupeptin, and 1 μM pepstatin A) for 30 min at 4°C. The lysates were centrifuged at 12,000 rpm for 20 min at 4°C and the supernatants were immunoprecipitated using anti-FLAG M2 Affinity Gel (Sigma-Aldrich) overnight at 4°C. After washing the gel four times with TNE buffer, the FLAG-tagged CADM4 was eluted using 150 μg/mL of 3 × FLAG peptide (ApexBio, Houston, TX, USA) and subjected to Western blotting. The CADM1-GFP, E-cadherin-GFP, and CADM4-FLAG expression vectors were previously described (Kuramochi et al., 2001; Williams et al., 2006; Sakurai-Yageta et al., 2015). The CADM3-GFP expression vector was obtained by cloning *CADM3* cDNA into the pEGFP-N3 BglII-SalI site (TaKaRa Bio Inc.).

### RNA interference

CADM1 was knocked down by the lentiviral delivery of short-hairpin (sh)RNA. The oligonucleotides for shRNA against CADM1 or non-targeting shRNA were annealed and subcloned into the pENTR4-H1 BglII-XbaI site (RIKEN BioResource Center), and the fragment that included the H1 promoter and shRNA precursor was transferred to the CS-RfA-CG destination vector (RIKEN BioResource Center) using LR clonase (Thermo Fisher Scientific). The obtained shRNA-expressing lentiviral vector as well as pCAG-HIVgp and pCMV-VSV-G-RSV-Rev (RIKEN BioResource Center) were co-transfected into the 293FT cells using Polyethylenimine Max (Polysciences, Warrington, PA, USA). After 72 h, the culture supernatants containing the lentiviruses were collected, and the viruses were concentrated by ultracentrifugation at 50,000 × g for 2 h at 4°C. The virus titers were determined using qPCR Lentivirus Titration Kit (Applied Biological Materials, Richmond, Canada), SYBR Green PCR Master Mix (Applied Biosystems, Foster City, CA, USA), and the ABI 7300 real-time PCR system (Applied Biosystems). ATN1 cells were infected with the lentiviruses at a multiplicity of infection of 5. The cells expressing shRNA were GFP-positive cells obtained using FACS Aria II (BD Biosciences, Franklin Lakes, NJ, USA). The target sequences of shRNA were as follows: shCADM1, 5,5′-CGAAAGACGTGACAGTGAT-3′; shCADM1, 8,5′-GCGCTTGAGTTAACATGT-3'; and shControl, 5'-ACTACCGTTGTTATAGGT-3′.

### Western blotting

Western blotting was performed as previously described (Ito et al., 2011). Briefly, cell lysates were prepared using a lysis buffer (50 mM Tris-HCl [pH 7.5], 150 mM NaCl, 1 mM EDTA, 1% Triton X-100) containing a protease inhibitor cocktail. Equal amounts of total protein were fractionated in 7.5% SDS-PAGE, transferred to a polyvinylidene difluoride membrane (Merck Millipore), and incubated with primary antibodies. Primary antibody binding was detected using the Pierce Western Blotting Substrate (Thermo Fisher Scientific) with horseradish peroxidase-conjugated secondary antibodies, and the signals were visualized using ImageQuant LAS 4000 mini (GE Healthcare).

### Cell-spreading assay

A cell-spreading assay was performed as previously described (Murakami et al., 2014). Coverslips were precoated with 50 μg/mL poly-L-lysine (Sigma-Aldrich), fixed with 0.5% glutaraldehyde (Sigma-Aldrich), and then coated with 7 μg/mL CADM4-Fc or normal human IgG (Sigma-Aldrich) in PBS. After blocking with 1% BSA (Wako Pure Chemical Industries) in HBSS (Thermo Fisher Scientific), 1 × 10^4^ cells in culture medium were plated onto coverslips and incubated for 60 min at 37°C. The cells were fixed with 4% paraformaldehyde and labeled with Alexa Fluor 488 Phalloidin (Thermo Fisher Scientific). The coverslips were mounted with Prolong Gold antifade reagent with DAPI (Thermo Fisher Scientific), and the cells were imaged using the Axio Observer D1 epifluorescence microscope (Carl Zeiss, Oberkochen, Germany). The cell area was measured using AutoMeasure, AxioVision Version 4 (Carl Zeiss). An area comprising 100 cells was examined in each experiment. Statistical significance was determined using Student's *t*-test.

## Results

### Extracellular interactions between the CADM and nectin family molecules

**Figure 1:**
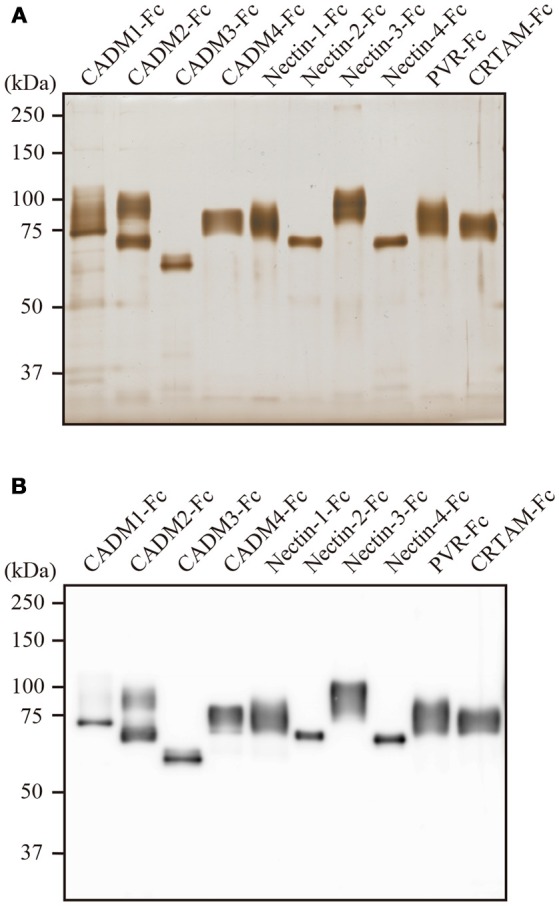
Characterization of the purified IgSF-Fc proteins. **(A)** Silver staining of the purified IgSF-Fc proteins. Each 100 ng of IgSF-Fc protein was fractionated in a 7.5% SDS-PAGE gel. **(B)** Western blotting of the purified IgSF-Fc proteins using an anti-human IgG-Fc antibody.

The CADM and the Nectin family of IgSF cell-adhesion molecules contribute to cell–cell adhesion through homophilic and heterophilic interactions. We thus analyzed the interactions among IgSF molecules, focusing on the CADM and the Nectin family members and their related molecules using SPRi. Extracellular domains of 10 IgSF molecules, including the CADM family (CADM1–CADM4), Nectin family (Nectin-1–Nectin-4), PVR, and CRTAM, were produced as Fc-fused proteins in the supernatant of transfected 293-F cells. In SDS-PAGE, the purified proteins exhibited a shift toward higher molecular weight after their post-translational modifications such as glycosylation as observed in human tissues (Figure 1).

**Figure 2:**
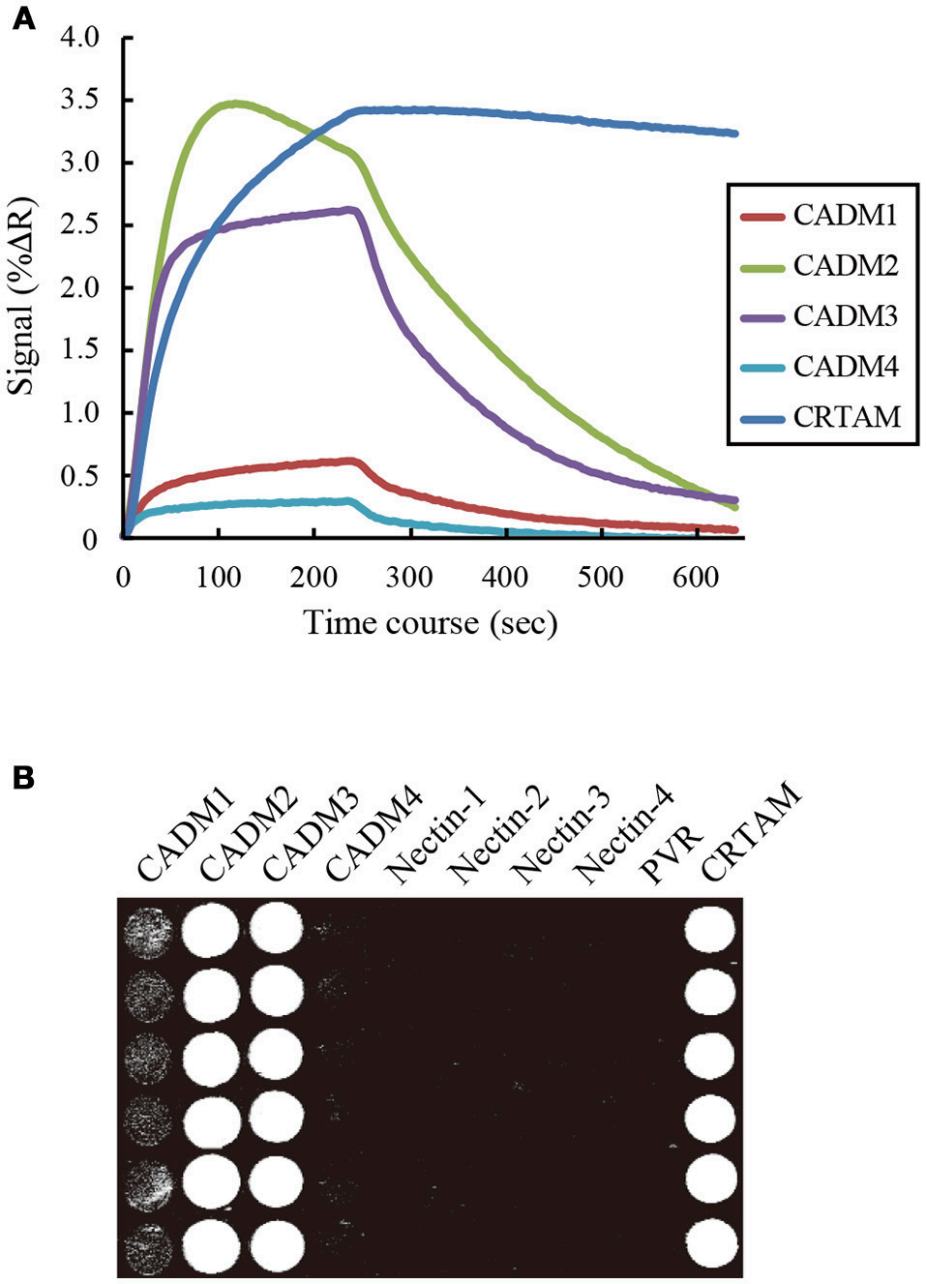
Surface plasmon resonance imaging analysis of the interactions between CADM1 and its binding partners. **(A)** SPRi analysis was performed by injecting 2.4 μM CADM1-Fc for 4 min onto the sensor chip where the 10 IgSF-Fc proteins were spotted. The analysis was performed in hexaplicate and the averaged signal of the six spots are shown. Only five molecules giving positive signals are shown. **(B)** The percent change in reflectivity (%ΔR) at 60 s was visualized as a bright spot on the image.

To examine the interactions between CADM1 and the 10 IgSF molecules using SPRi, IgSF-Fc proteins were prepared at a concentration of 7–24 μM as described in Supplementary Table 1 and spotted on an SPR sensor chip in hexaplicate. When CADM1-Fc was subjected to SPRi, the sensorgrams as well as the SPR image showed that CADM1 interacts with CADM1, CADM2, CADM3, and CRTAM (Figures 2A,B). Furthermore, the sensorgrams and SPR image demonstrated that CADM1 interacts with CADM4, which had not been reported previously. The association rate constants (*k*_*on*_), dissociation rate constants (*k*_*off*_), and dissociation constants (*K*_*d*_) of the interactions were then calculated from the sensorgrams (Table 1). Notably, the response curve of CRTAM was quite different from that of the other molecules. The dissociation of CADM1 from CRTAM was much slower than that from the CADM family proteins with approximately a 100-fold difference in *k*_*off*_ (58–173), suggesting that the interaction between CADM1 and CRTAM is stable and does not easily dissociate once it is formed.

**Table 1:** Surface plasmon resonance analysis of the interaction between CADM1 and its binding partners.

**Analyte protein**	**Immobilized protein**	***k_*on*_* (M^−1^s^−1^)**	***k_*off*_* (s^−1^)**	***K_*d*_* (nM)**
CADM1-Fc	CADM1-Fc	6.4 × 10^4^	7.3×10^−3^	114.4
CADM1-Fc	CADM2-Fc	7.4 × 10^4^	6.4×10^−3^	86.8
CADM1-Fc	CADM3-Fc	8.4 × 10^4^	7.0×10^−3^	80.1
CADM1-Fc	CADM4-Fc	9.9 × 10^4^	1.9 × 10^−2^	192.7
CADM1-Fc	CRTAM-Fc	3.1 × 10^4^	1.1×10^−4^	3.3

**Figure 3:**
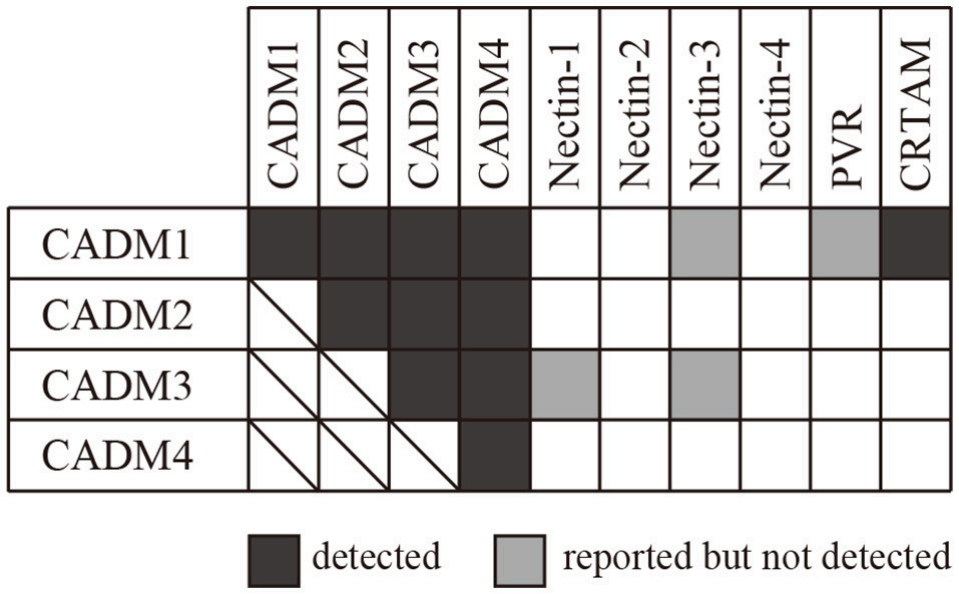
Combination of the interactions between the CADM family proteins and the 10 IgSF molecules including the CADM and the Nectin family proteins. The interactions detected by SPRi are shown in black and those reported but not detected by SPRi are shown in gray.

We then subjected CADM2-Fc, CADM3-Fc, and CADM4-Fc to SPRi and examined their possible interaction with the 10 IgSF-Fc proteins. These analyses detected both the homophilic and heterophilic interactions of CADM2, CADM3, and CADM4 with all the CADM family proteins (Figure 3). However, some of the interactions previously reported were not detected in the present SPRi analysis. These include the heterophilic interactions of CADM1 with Nectin-3 or PVR and the heterophilic interactions of CADM3 with the Nectins 1 or 3 (Shingai et al., 2003; Kakunaga et al., 2005; Wakayama et al., 2007; Figure 3). Failure of the detection of these known interactions by SPRi might be caused by their lower affinity than the detection limit of SPRi in this experimental condition. Alternatively, when interactions previously reported in cell-based assay required some accessory proteins, SPRi could not detect such interactions because it could only detect direct interactions between two molecules.

### Characterization of the interaction between CADM1 and CADM4

**Figure 4:**
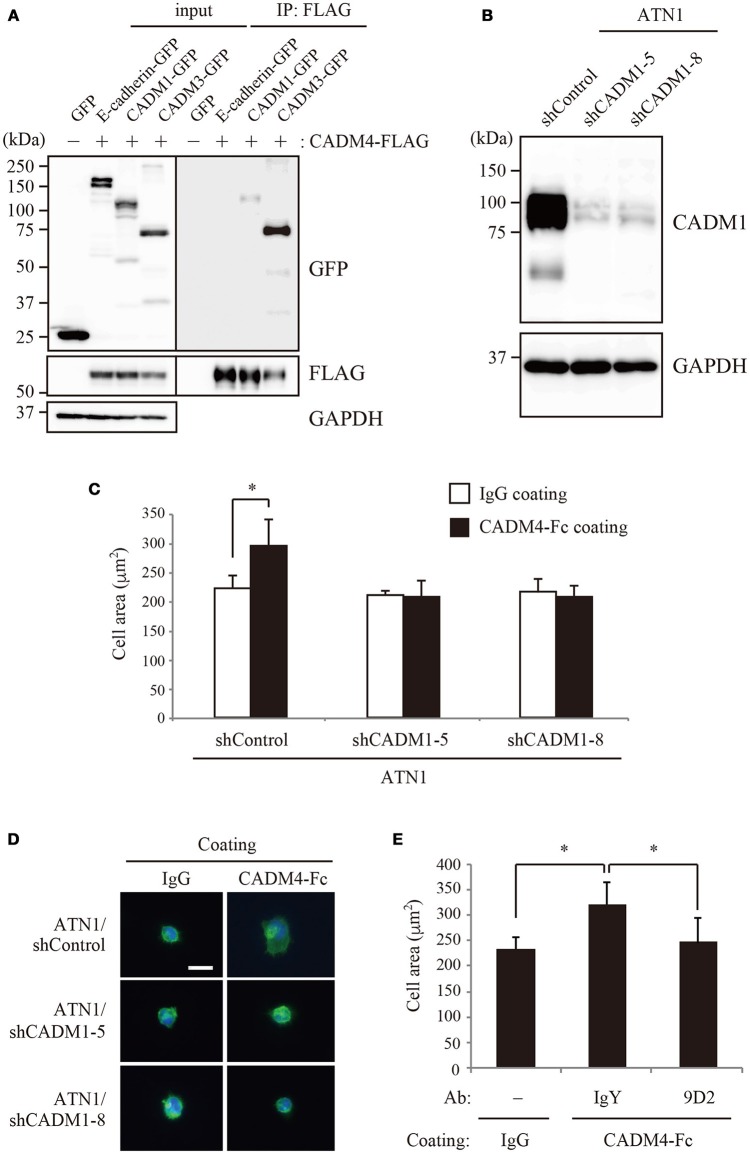
The interaction between CADM1 and CADM4 is involved in the cell spreading of ATN1 cells. **(A)** The *trans*-interaction between CADM1 and CADM4 in the extracellular region was examined by immunoprecipitation assay. E-cadherin and CADM3 were used as the negative and positive controls, respectively. **(B)** Knockdown of CADM1 in ATN1 cells by two independent shRNAs, shCADM1-5, and shCADM1-8. **(C)** ATN1 cells with control shRNA or shCADM1 were incubated on coverslips coated with control IgG or CADM4-Fc. The area of 100 cells was measured in an assay and the average of three independent experiments is shown. *p < 0.05 by *t*-test. **(D)** Representative images of the spread morphology of ATN1 cells with each shRNA incubated on IgG- or CADM4-Fc-coated glasses. The cells were visualized by staining the actin cytoskeleton with Alexa Fluor 488-labeled phalloidin. The nuclei were stained with DAPI. Magnification, × 200. Scale bar, 20 μm. **(E)** ATN1/shControl cells were incubated on IgG or CADM4-Fc for 60 min in the presence of control chicken IgY (10 μg/mL) or anti-CADM1 antibody, 9D2 (10 μg/mL). The average cell area of three independent experiments is shown. *p < 0.05 by *t*-test.

To examine the possible interaction between CADM1 and CADM4 in cell-based analyses, 293FT cells independently transfected with either CADM4-FLAG or CADM1-GFP were co-cultured and subjected to immunoprecipitation. The signal from CADM1-GFP was detected in the precipitates using an anti-FLAG antibody (Figure 4A), suggesting that CADM1 and CADM4 form a *trans*-heterophilic interaction with neighboring cells. Next, we examined the interaction between CADM1 and CADM4 in cell spreading assay using a cell line, ATN1, which is derived from adult T-cell leukemia (ATL) expressing a high amount of CADM1 protein. This assay can detect the interaction of CADM1 expressed in an ATL cell with purified CADM4-Fc protein immobilized on the glass. ATN1 cells show CADM1-dependent morphological spreading when incubated on endothelial cells or fibroblasts (Masuda et al., 2010). Similarly, when incubated on CADM4-Fc-coated glass, ATN1 cells exhibited spread morphology with increased cell area. In contrast, CADM1 knockdown by two independent shRNAs inhibited the spreading of ATN1 cells on the CADM4-Fc-coated glass (Figures 4B–D). Furthermore, a neutralizing CADM1 antibody blocked the spreading of ATN1 cells on CADM4-Fc-coated glass (Figure 4E). These results indicate that the heterophilic interaction between CADM1 and CADM4 induces the spreading of ATN1 cells. It is known that CADM1 promotes adhesion of ATL cells to vascular endothelial cells (Sasaki et al., 2005; Masuda et al., 2010). However, the partners for CADM1 binding onto endothelial cells have not been identified yet. Since CADM4 expression is observed on endothelial cells (Yamana et al., 2015), the *trans*-heterophilic interaction between CADM1 and CADM4 might be involved in ATL cell adhesion to endothelial cells.

## Discussion

In the present study, we developed a platform for multiple and sensitive detection of extracellular protein–protein interactions using an SPRi system. In comparison with other high-throughput techniques for detecting protein–protein interactions such as protein microarrays or AVEXIS, the throughput of SPRi might be limited because SPRi requires a large amount of purified proteins. In fact, protein microarrays are mainly designed for screening interacting partners from a large-scale of protein library. For example, a commercially available protein microarray (ProtoArray, Thermo Fisher Scientific) contains more than 9,000 proteins immobilized on a glass slide. By contrast, SPRi have a great advantage in detecting protein–protein interaction sensitively and quantitatively on the basis of the reaction rate constants of the interactions. This advantage is attributed to the unique detection principle of SPRi, in which protein–protein interaction is quantitatively monitored in real time on the basis of the changes in the intensity of reflected light with attenuation by SPR phenomena. Thus, SPRi is more sensitive in detecting the molecular interactions than protein microarrays or AVEXIS, which monitors direct interaction of two molecules by fluorescence- or chemiluminescence-based techniques. As to the instruments, SPRi and protein microarrays need dedicated detectors as well as microarray spotters if customizing protein arrays are necessary, whereas AVEXIS is relatively a low-cost system except for a microplate reader.

In this study, we focused on CADM family proteins and examined possible protein–protein interactions among 10 IgSF molecules, including CADMs 1–4 and their structurally related 6 proteins, using an SPRi system. Then, we newly identified an interaction between CADM1 and CADM4, although previous analyses using affinity chromatography (Fogel et al., 2007) or cell-adhesion assay (Maurel et al., 2007; Thomas et al., 2008) could not detect this interaction. The discrepant results suggest that CADM1–CADM4 interaction might not provide enough activity of cell adhesion in the previous study. On the other hand, several interactions reported by previous studies were not detected in the present study. These include interactions between CADM1–Nectin-3, CADM1–PVR, CADM3–Nectin-1, and CADM3–Nectin-3, which were detected essentially by cell-based assay (Kakunaga et al., 2005; Wakayama et al., 2007; Masuda et al., 2010). This discrepancy may be caused if previously detected interactions are only formed in the presence of other accessary interactions between unidentified molecules in cell-based assay. Alternatively, we might detect these interactions by increasing the concentration of proteins in SPRi analysis, because extraordinary high concentration of analyte protein (>44 μM) was used to detect homophilic interactions between Nectin family proteins in the previous study (Harrison et al., 2012).

In SPRi analysis, we had the privilege of analyzing the kinetics of the interaction between CADM1 and each binding partner. Among them, the kinetics of the interaction between CADM1 and CRTAM was quite different from that between CADM1 and CADMs 1–4 (Figure 2). In particular, the *k*_*off*_ of the interaction between CADM1 and CRTAM is much smaller than those of CADM1 and CADMs 1–4 (Table 1). Small *k*_*off*_ indicated that the interaction between CADM1 and CRTAM is stable and dissociates very slowly. In fact, this interaction is found physiologically between epithelial cells expressing CADM1 and CD8^+^ T or natural killer (NK) cells expressing CRTAM (Arase et al., 2005; Boles et al., 2005; Galibert et al., 2005). Stable interaction with very slow dissociation would be suitable for CD8^+^ T or NK cells to exhibit efficient cytotoxic effects on epithelial target cells expressing CADM1 through specific cell–cell adhesion. On the other hand, homophilic interaction of CADM1 has a large *k*_*off*_, which makes it more susceptible to dissociation. Therefore, CADM1 appears to be engaged in cell–cell adhesion with continuous association–dissociation processes. This is consistent with a previous report by fluorescence recovery after photobleaching, demonstrating continuous renewal of CADM1 on the plasma membrane of epithelial cells even in a static state (Sakurai-Yageta et al., 2015). The difference in the kinetics of each interaction appears to be caused by the difference in the higher order structures of the molecules. The binding mode between CADM1 and CRTAM is a double “lock-and-key” structure (Zhang et al., 2013), whereas the homophilic binding of CADM1 appears to be mediated by a single hydrophobic interaction (Dong et al., 2006).

In this study, the *K*_*d*_ of the interaction between CADM1-Fc and CRTAM-Fc was estimated to be 3.3 nM. This constant was obtained by SPRi analysis using the whole extracellular domains fused with Fc that were purified from 293-F cells. On the other hand, the *K*_*d*_ of the interaction between the IgV domain of CADM1 and that of CRTAM purified from *E. coli* was 12.5 μM with *k*_*on*_ = 6.27 × 10^2^ M^−1^s^−1^ and *k*_*off*_ = 7.84 × 10^−3^ s^−1^ in a previous study (Zhang et al., 2013). The marked difference in the *K*_*d*_ between two experiments suggest that the IgC-loops of CADM1 and CRTAM facilitate a part of interaction, although the N-terminal IgV-loops of each molecule are primarily involved in the interaction (Arase et al., 2005). In addition, dimerization by the Fc-tag used in our study may mimic and enhance the *cis*-dimer formation of both CADM1 and CRTAM (Masuda et al., 2002; Shingai et al., 2003; Arase et al., 2005). Moreover, the *N*-glycosylation of CADM1 and CRTAM which are only observed in our analysis using 293-F cells but not in the study using *E. coli* would enhance the interaction between CADM1 and CRTAM as was shown in the interaction between CADM1 and CADM2 (Fogel et al., 2010). These findings suggest that *K*_*d*_ obtained in our SPRi analysis would reflect the physiological condition more correctly.

Using SPRi analysis, we newly identified an interaction between CADM1 and CADM4. Furthermore, we showed that CADM1–CADM4 interaction was involved in the cell spreading activity of an ATL cell line, ATN-1 (Figure 4). CADM1 is ectopically expressed in ATL and promotes tumor growth and infiltration (Sasaki et al., 2005; Dewan et al., 2008), whereas CADM4 is expressed in endothelial cells. Therefore, interaction of CADM1 in ATL cells with CADM4 in endothelial cells may trigger infiltration of ATL cells into various organs. As this case illustrates, our platform using the SPRi system has the potential to identify a low-affinity but physiologically significant molecular interactions that have not been detected by other assays. Taking into account that SPR can determine the kinetics of the on and off rates for molecular interactions, our platform would provide an excellent and useful tool for both detecting and characterizing extracellular protein–protein interactions.

## Author contributions

TI and SM conceived and designed the experiments. YKa and YKu performed vector construction, protein production and immunoprecipitation. DS performed the cell spreading assay. MO-N produced CADM1-knockdown cells. DI performed the SPR analyses. TI and YM wrote the manuscript.

### Conflict of interest statement

DI and SM are employees of HORIBA Ltd., which is the company that sells the surface plasmon resonance imaging system used in this study. The remaining authors declare that the research was conducted in the absence of any commercial or financial relationships that could be construed as a potential conflict of interest.

## Abbreviations

SPRi: surface plasmon resonance imaging
IgSF: immunoglobulin superfamily
CRTAM: class-I MHC-restricted T-cell-associated molecule
Fc: fragment crystallizable region
ATL: adult T-cell leukemia.
