# Supplementary material for: Quantitative Analysis of Interaction Between CADM1 and Its Binding Cell-Surface Proteins Using Surface Plasmon Resonance Imaging
Source: Front Cell Dev Biol. 2018 Aug 7;6:86. doi: 10.3389/fcell.2018.00086 (PMC6090299; doi:10.3389/fcell.2018.00086)
Supplement: Supplementary file 1 [file Presentation_1.PDF]

Supplementary Material

Quantitative analysis of interaction between CADM1 and its binding cell-surface proteins using surface plasmon resonance imaging

Takeshi Ito, Yutaka Kasai, Yuki Kumagai, Daisuke Suzuki, Misaki Ochiai-Noguchi, Daisuke Irikura, Shiro Miyake, Yoshinori Murakami*

*** Correspondence:** Corresponding Author: ymurakam@ims.u-tokyo.ac.jp

1 Supplementary Figures and Tables

1.1 Supplementary Figures

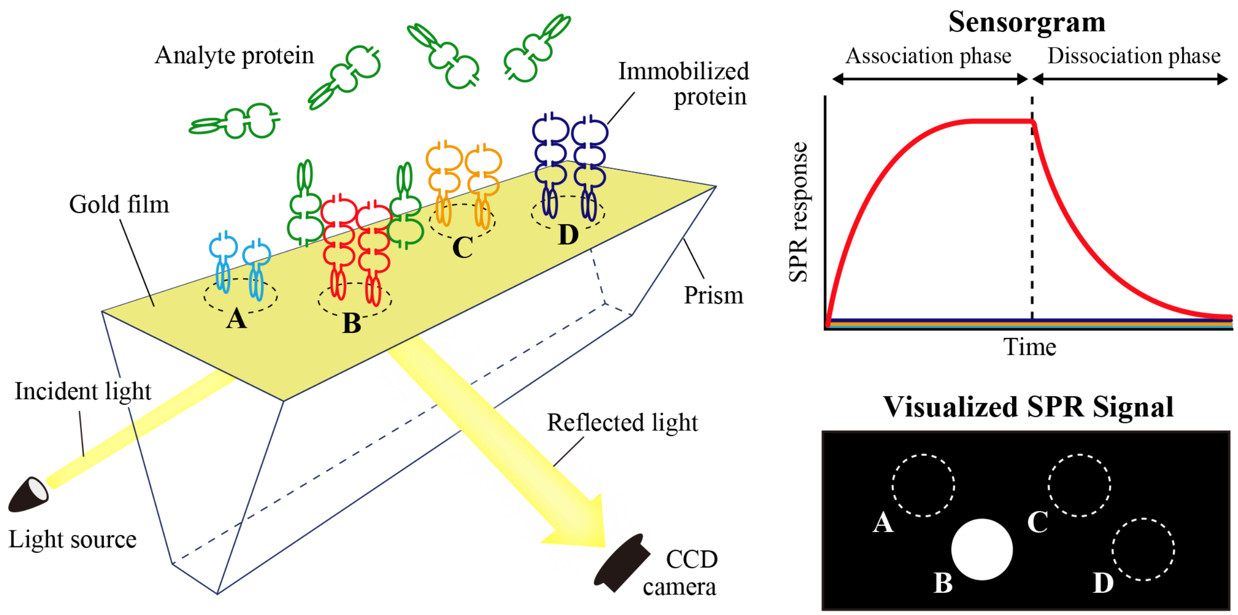

Supplementary Figure 1. Experimental scheme of surface plasmon resonance imaging (SPRi) analysis. Proteins were spotted and immobilized on an SPR biochip consisting of a prism whose surface was coated with gold film. The binding of analyte protein to the immobilized protein was detected as a shift of SPR angle, which was recorded by a CCD camera. This resonance angle shifting was visualized as sensorgrams and bright spot images.

1.2 Supplementary Tables

Supplementary Table 1. Concentration of the Fc-fusion proteins used for the surface plasmon resonance imaging analysis.

Protein	Concentration (mg/mL)	Molecular weight (kDa)	Molar concentration (μM)
CADM1-Fc	0.9	72	13
CADM2-Fc	2	85	24
CADM3-Fc	0.8	60	13
CADM4-Fc	1.3	75	17
Nectin-1-Fc	1.1	75	15
Nectin-2-Fc	0.5	70	7
Nectin-3-Fc	1.7	100	17
Nectin-4-Fc	1.2	68	18
PVR-Fc	1.2	80	15
CRTAM-Fc	1.1	75	15
